# Periodontal disease, peri-implant disease and levels of salivary biomarkers IL-1β, IL-10, RANK, OPG, MMP-2, TGF-β and TNF-α: follow-up over 5 years

**DOI:** 10.1590/1678-7757-2018-0316

**Published:** 2019-02-21

**Authors:** Alex Martins GOMES, Dhelfeson Willya Douglas-de-OLIVEIRA, Sérgio Diniz FERREIRA, Tarcília Aparecida da SILVA, Luís Otávio Miranda COTA, Fernando Oliveira COSTA

**Affiliations:** 1 Universidade Federal de Minas Gerais, Faculdade de Odontologia, Departamento de Periodontia, Belo Horizonte, Minas Gerais, Brasil.; 2 Clinica particular, Belo Horizonte, Minas Gerais, Brasil.

**Keywords:** Periodontitis, Peri-implant mucositis, Saliva, Biomarkers, Cytokines

## Abstract

**Objective:**

The aim of this study was to evaluate the levels of salivary biomarkers IL-1β, IL-10, RANK, OPG, MMP-2, TG-β and TNF-α in individuals with diagnosis of peri-implant mucositis in the absence or presence of periodontal and peri-implant maintenance therapy (TMPP) over 5 years.

**Material and Methods:**

Eighty individuals diagnosed with peri-implant mucositis were divided into two groups: one group that underwent periodontal and peri-implant regularly maintenance therapy, called GTP (n=39), and a second group that received no regular maintenance GNTP (n=41). Each participant underwent a complete periodontal and peri-implant clinical examination. Collection of saliva samples and radiographic examination to evaluate peri-implant bone levels were conducted at two times: initial examination (T1) and after 5 years (T2). The salivary samples were evaluated through ELISA for the following markers: IL-1β, IL-10, RANK, OPG, MMP-2, TGF and TNF-α.

**Results:**

A higher incidence of peri-implantitis was observed in the GNTP group (43.9%) than in the GTP group (18%) (p=0.000). All individuals (n=12) who presented peri-implant mucositis and had resolution at T2 were in the GTP group. After 5 years, there was an increase in the incidence of periodontitis in the GNTP group compared to the GTP group (p=0.001). The results of the study revealed an increase in the salivary concentration of TNF-α in the GNTP group compared to the GTP group. The other salivary biomarkers that were evaluated did not show statistically significant differences between the two groups.

**Conclusions:**

The salivary concentration of TNF-α was increased in individuals with worse periodontal and peri-implant clinical condition and in those with a higher incidence of peri-implantitis, especially in the GNTP group. Longitudinal studies in larger populations are needed to confirm these findings and elucidate the role of this biomarker in peri-implant disease.

## Introduction

The infectious-inflammatory disease occurring around implants is known as peri-implant disease (PID) and it may manifest itself as peri-implantitis mucositis or periimplantitis. Peri-implant mucositis (MP) is characterized by inflammatory infectious disease that results in reversible inflammation of peri-implant soft tissues and peri-implantitis by the loss of soft and hard tissues around the implants.^1^

Studies have attempted to clarify the role of cytokines in this immuno-inflammatory response, but the literature presents conflicting and scarce data regarding the concentration of potential markers of periodontitis (PE) and peri-implantitis^2^ ^,^ ^3^ (PI) and their role in the progression of these diseases.^4^ ^,^ ^5^ In addition, cytokines, chemokines, enzymes of cellular destruction and the molecules produced as a consequence of tissue destruction in PE and PID are released and can be identified in saliva.^6^ ^,^ ^7^

Recent studies indicate that the use of immunological markers may aid in the diagnosis of health and PID. The advantage of using saliva instead of blood or gingival crevicular fluid analysis is that it is a non-invasive collection method, has high availability, is painless and does not require special equipment for collection.^8^

It is a fact that the literature includes numerous studies on the association between PID and levels of inflammatory markers in peri-implant fluid sulcus (FSPI), in gingival tissue biopsies and blood. However, surprisingly, despite the advantages of using saliva, salivary marker studies related to the presence and progression of PID are rare. Additionally, to date, clinical changes in peri-implant conditions associated with salivary markers in individuals with MP in the absence or presence of regularly periodontal and peri-implant maintenance therapy (TMPP) have not been reported in longitudinal follow-up studies.

The literature highlights that TMPP decreases biological complications and increases the success of long-term implants.^9^ ^-^ ^11^. In this sense, the objective of this study was to compare the salivary concentrations of interleukin (IL) IL-1β, IL-10, RANK, OPG, matrix metalloproteinase (MMP) MMP-2, transforming growth factor beta (TGF- β) and tumor necrosis factor alpha (TNF-α) immunological markers related to the peri-implantation condition of individuals with MP between baseline examination and final exam in the absence and presence of TMPP.

## Material and methods

### Sample

The sample for this study was obtained from a study conducted in 2006 with partially edentulous individuals rehabilitated with dental implants having the objective of identifying possible risk factors and the prevalence of PID.^12^ Eighty nonsmokers who were diagnosed with MP in 2006 (T1) were re-called annually to TMPP, and new periodontal/peri-implant clinical exams and immunological collections were repeated in 2012 (T2), resulting in a 5-year interval between T1 and T2. These individuals were divided into 2 groups related to regularity TMPP: one group that performed TMPP regularly, that is, at least one visit/year (GTP=39), and one that did not perform TMPP regularly, that is, less than one visit/year (GNTP=41). The periodontal and peri-implant clinical data of these individuals were previously reported.^13^

In T1 and T2, the following parameters for the teeth and implants in periodontal/peri-implant examinations were recorded: clinical attachment level (CAL), periodontal probing depth (PS) and peri-implant probing depth (PSi), bleeding on periodontal probing (BOP) and bleeding on peri-implant probing (BOPi), periodontal (PL) and peri-implant (PLi) plaque index.^14^ ^,^ ^15^ In the implants, the presence of peri-implant suppuration (Si) was also evaluated, and radiographic measurements were also conducted to evaluate bone levels. The methodology for collecting these clinical data was described in detail before.^12^ Salivary sample collection was performed at the time of clinical evaluations at T1 and T2 and will be described later.

The procedure and the research were explained in detail to each participant, and free and informed consent was obtained. Additionally, all 80 individuals evaluated for periodontal and peri-implant parameters were referred for free periodontal/peri-implant maintenance treatment in each scheduled visit. This study was approved by the Research Ethics Committee under protocol number 05650203000-10.

### Inclusion and exclusion criteria

This study adopted the same inclusion and exclusion criteria as the original data published.^12^ To be included in the sample, the participants could not have systemic diseases that influence periodontal and peri-implant clinical examination, had to attend the annual scheduled visit for the TMPP, in GTP group, had not used systemic antimicrobial medicine in the 3 months prior to clinical examination, and had unitary or partial prosthetic rehabilitations suitable for a correct clinical examination. Patients with prosthetic overdentures (due to the high incidence of soft tissue complications and difficulties during the exam) were excluded. Smokers (individuals who smoked more than 100 cigarettes in their lifetime) and ex-smokers (individuals who quit smoking up to 3 years before the clinical exams) were excluded from the study.^11^ ^,^ ^16^ All evaluated implants had at least 6 months and up to 5 years in function.

### Diagnosis of periodontal and peri-implant diseases

The PE was diagnosed with presence of 4 or more teeth with one or more sites having PS>4 mm and CAL≥3 mm at the same site.^17^ The implant/subject was diagnosed with MP in the presence of a site with BOPi.^18^ An implant/subject was diagnosed with PI when BOPi and/or Si, PSi≥5 mm and presence of bone loss confirmed by radiography^19^ or a value of PSi≥5 mm, even though there was no SSi and/or Si, howeber showing bone loss at the radiographic examination.^12^ If the individual had an implant diagnosed with PI, then another with MP was considered the worst diagnosis.

### Collection of saliva samples for immunological analysis

#### Salivary exam

Non-stimulated total saliva samples were collected by a single investigator (F.O.C.) whenever possible, at the same time in the two-hour period after the last meal. The participants were instructed to rinse their mouths with water, and 5 ml of the saliva produced was collected in a Falcon-type millimeter tube. Saliva samples were frozen at -80°C until analysis by an investigator (T.A.S.) who was unaware of the previous phases of the experiment. For the assays, the samples were thawed and diluted 1:1 in a solution of PBS (0.4 mM NaCl and 10 mM NaPO4) containing protease inhibitors (0.1 mM phenylmethylsulfonyl fluoride, 1 mM benzethonium chloride, 10 mM EDTA and 0.01 mg/ml aprotinin A) and 0.05% Tween-20. The samples were later centrifuged at 3000 rpm for 15 minutes at 4°C, and the supernatant was used to analyze the concentrations of IL-1β, IL-10, MMP-2/TIMP-2 complex, RANK, OPG, TGF-β and TNF-α using commercially available kits (R & D Systems, Minneapolis, MN, USA). Biomarker concentrations were expressed in pg/ml according to the manufacturer’s specifications and normalized to total saliva´s proteins at the collection times (T1 and T2).

## Statistical analysis

The data collected were analyzed with the application SPSS (Statistical Package for Social Sciences, IBM Inc.Chicago, Ilinois, USA) version 23.0. Initially, descriptive analyses were performed to obtain the mean, standard deviation, absolute and relative frequency of the data. The normality of the data was verified by the Kolmogorov-Smirnov test. To verify if there were differences in the variables investigated between groups, the data were subjected to Mann-Whitney U and chi-square (or Fisher exact) tests. To verify if there was an association in the biomarker concentrations between the initial and final diagnosis, the data were subjected to the Wilcoxon test. It should be noted that for the analysis of data from T1 to T2, the GTP and GNTP groups were each subdivided into 3 subgroups according to peri-implant status in T2: health, MP and PI. The level of significance was set at 5% (p<0.05).

## Results

The diagnosis of periodontal and peri-implant disease of the sample at T1 and T2 are presented in Table 1. There was an increase in the number of individuals with PE in the GNTP group when comparing T1 (22.0%) and T2 (41.5%) (Table 1). In the GNTP group, individuals with MP had lower levels of TNF-α when compared to individuals with PI (p=0.033). There was no statistically significant difference in the concentrations of the other markers evaluated between T1 and T2 (Table 2). No significant difference was found in the concentrations of the biomarkers evaluated for individuals with MP diagnosis in T1 and T2. Additionally, no significant difference was found in the concentrations of the biomarkers evaluated for the individuals with the MP diagnosis at T1 and PI at T2 (Table 3). In addition, all individuals (n=12) who manifested MP at T1 but were presented as healthy at T2 were in the GTP group (Table 4). None of the biomarkers evaluated had significantly different concentrations between healthy and MP implants.

**Table 1:** Sample characteristics

Variants	Baseline exam (T1)			Final exam (T2)		
	GNTP n = 41	GTP n = 39	p	GNTP n = 41	GTP n = 39	p
Periodontal diagnosis						
Healthy	32 (78.0%)	29 (74.4%)	0.698	24 (58.5%)	28 (71.8%)	0.214
PE	9 (22.0%)	10 (25.6%)		17 (41.5%)	11 (28.2%)	
Peri-implant diagnosis						
Healthy	0	0	NA	0 (0.0%)	12 (30.7%)	0
MP	41	39	NA	23 (56.0%)	20 (51.2%)	
PI	0	0	NA	18 (43.9%)	7 (18%)	

GNTP: group without periodontal/peri-implant preventive maintenance; GTP: group with periodontal/peri-implant preventive maintenance. PE: periodontitis; MP: peri-implant mucositis; PI: peri-implantitis; NA: not applicable

**Table 2:** Comparison of biomarker concentrations (pg/ml) in GNTP and GTP groups related to clinical diagnose at final exam

	GNTP			GTP			TOTAL SAMPLE		
	MP	PI		MP	PI		MP	PI	
	MEAN (SD)	MEAN (SD)	p	MEAN (SD)	MEAN (SD)	p	MEAN (SD)	MEAN (SD)	p
IL-1β	22630.3 (24724.1)	19592.4 (22324.7)	0.729	20137.2 (26300.9)	102081.9 (217171.7)	0.240	21470.7 (25193.4)	42689.5 (116502.6)	0.727
IL10	4.7 (12.8)	8.5 (20.2)	0.669	4.3 (9.0)	4.8 (9.5)	0.766	4.5 (11.13)	7.4 (17.7)	0.612
TNF-α	**2.2 (10.8)**	**14.2 (24.4)**	**0.033**	-	2.2 (5.9)	-	**1.2 (7.9)**	**10.9 (21.5)**	**0.005**
TGF-β	1.4 (4.3)	5.5 (15.5)	0.920	10.8 (13.0)	23.7 (36.6)	0.978	5.8 (10.4)	10.6 (24.0)	0.612
MMP-2	27.4 (34.7)	54.2 (121.8)	0.446	7.9 (5.0)	7.3 (5.0)	0.725	18.4 (27.2)	41.1 (104.7)	0.312
RANK	38.3 (10.7)	33.9 (9.8)	0.074	2.0 (5.0)	-	-	21.4 (20.2)	24.4 (17.6)	0.901
OPG	32.2 (44.3)	25.0 (27.4)	0.386	8.2 (9.6)	6.4 (4.2)	0.766	21.0 (34.9)	19.8 (24.7)	0.990

GNTP: group without periodontal/peri-implant maintenance; GTP: group with periodontal/peri-implant maintenance. IL: Interleukin; TNF: tumor necrosis factor alpha; TGF: transforming growth factor; MMP: metalloproteinase; RANK: Receptor activator of nuclear factor kappa; OPG: Osteoprotegerin MP: peri-implant mucositis; PI: peri-implantitis; SD standard deviation

**Table 3:** Comparison of biomarker concentrations (pg/ml) based on clinical evolution from initial examination(T1) to final exam (T2)

	MP			PI		
	T1 (MP)	T2 (MP)		T1 (MP)	T2 (PI)	
	Mean (SD)	Mean (SD)	p	Mean (SD)	Mean (SD)	p
IL-1β	17797.7 (25025.3)	21470.7 (25193.4)	0.217	21468.0 (23332.9)	42689.5 (116502.6)	0.506
IL-10	4.4 (11.7)	4.5 (11.1)	0.678	8.2 (18.3)	7.4 (17.7)	0.866
TNF-α	-	1.2 (7.9)	-	12.4 (22.7)	10.9 (21.5)	0.999
TGF-β	9.9 (24.6)	5.8 (10.4)	0.661	5.4 (9.4)	10.6 (24.0)	0.386
MMP-2	14.8 (14.3)	18.4 (27.2)	0.673	25.4 (39.3)	41.1 (104.7)	0.677
RANK	22.2 (18.7)	21.4 (20.2)	0.554	26.4 (15.1)	24.4 (17.6)	0.357

GNTP: group without periodontal/peri-implant maintenance; GTP: group with periodontal/peri-implant maintenance. IL: Interleukin; TNF: tumor necrosis factor; TGF: transforming growth factor; MMP: metalloproteinase; RANK: Receptor activator of nuclear factor kappa; OPG: Osteoprotegerin; n: individuals; MP: peri-implant mucositis; PI: peri-implantitis; SD standard deviation

**Table 4:** Comparison of biomarker concentrations (pg/ml) in the individuals in the GTP groups with healthy implants diagnosed at final exam(T2)

	MP(T1)	Healthy(T2)	PI (T2)	
		Mean (SD)	Mean (SD)	p*
IL-1	20137.28 (26300.93)	13655.44 (24495.43)	102081.93 (217171.72)	0.179
IL-10	4.38 (9.04)	1.87 (6.47)	4.88 (9.56)	0.523
TNF-α	-	-	2.23 (5.91)	-
TGF-β	10.90 (13.04)	8.82 (8.45)	23.78 (36.70)	0.998
MMP-2	7.98 (5.03)	6.16 (2.24)	7.34 (5.09)	0.704
RANK	2.05 (5.05)	1.25 (4.35)	-	0.532
OPG	8.24 (9.68)	10.82 (12.37)	6.48 (4.21)	0.860

GNTP: group without periodontal/peri-implant maintenance; GTP: group with periodontal/peri-implant maintenance. IL: Interleukin; TNF: tumor necrosis factor; TGF: transforming growth factor; MMP: metalloproteinase; RANK: Receptor activator of nuclear factor kappa; OPG: Osteoprotegerin; T1: initial examination; T2: final exam; n: individuals; MP: peri-implant mucositis; PI: peri-implantitis; SD standard deviation

## Discussion

Saliva samples can be easily obtained in a non-invasive manner and at low cost, but few published studies have examined saliva biomarkers to investigate the presence and progression of PID. In this sense, this longitudinal study aimed to evaluate peri-implant clinical condition and levels of salivary biomarkers IL-1β, IL-10, RANK, OPG, MMP-2, TGF and TNF-α in individuals diagnosed with MP in the presence or absence of TMPP over a 5-year period. After investigation of salivary levels, we observed that the concentration of TNF-α was significantly higher in individuals in the GNTP group who developed PI after 5 years. Our findings are in agreement with those of several authors who found increased salivary cytokine levels in cases of PE^18^ ^,^ ^20^ ^,^ ^21^ and PID.^22^ Another aspect of PID and cytokines was the focus of one study.^23^ The authors showed that after measuring the concentration of IL-1β in total saliva, saliva of the parotid gland and FSPI, only the FSPI measurement showed an increase in concentration when comparing a group of implants diagnosed with MP with a second group of implants diagnosed with PI. The authors attributed the results to the dilution of the mediators in the saliva that makes them difficult to detect. However, the sample consisted of only 20 implants with a cross-sectional analysis of cytokine concentrations. The present study included a sample of 80 implants and a 5-year follow-up, which may influence the contradictory results. One study^22^ reinforces the results of the present study because the authors evaluated 50 individuals and showed a higher concentration of IL-1β in unstimulated saliva in individuals with implants diagnosed with PI compared to healthy implants.

Notably, the results of our study showed that the individuals who had resolved MP with peri-implant health belonged to the GTP group, and the prevalence of PI in the GNTP group (43.9%) was higher than in the GTP group (18%) (p<0.001). Similar findings were found in the literature^24^, one study^25^ reported that in 47 individuals with a history of PE followed for 7.9 years, the prevalence of PI was of 31.9% among the participants who underwent TMPP and 52.2% among those who did not undergo TMPP (p=0.102). The authors noted that lack of adherence to a regular periodontal/peri-implant maintenance program is correlated with a high incidence of peri-implant bone loss and implant loss. Hence, three main factors may have greatly influenced our findings: smoking, presence of PE and the absence of TMPP. Smoking is considered a major risk factor for PID.^19^ In this study, smokers were excluded from the sample in order to minimize this confounding factor in data analysis. In the GTP group, 12 individuals with MP in T1 were diagnosed as healthy at T2. This result is in agreement with studies on the influence of preventive maintenance in the control of PE and MP. The findings of recent systematic reviews and meta-analysis^14^ showed that TMPP is important in preventing and reducing the occurrence of PE and MP. Another systematic review study with meta-analysis stressed the importance of peri-implant maintenance, as it is the best way to prevent PI is MP control.^26^ The literature shows that professional follow-up in maintenance consultations prevents the development of PE, which is a risk factor for PI.^27^ Another study has shown that TMPP reduces the occurrence of PI in individuals with a history of PE, and the lack of TMPP is correlated with a higher incidence of peri-implant bone loss in individuals with and without a history of PE.^28^ According to the literature,^29^ the primary objective for avoiding the occurrence of complications with implants is based on a reinforcement of plaque control and reduction of risk factors, such as smoking and adjustment of prostheses, which hinder good local hygiene.

In addition to periodic maintenance, the concentrations of periodontal and peri-implant biomarkers have been studied in an attempt to predict their progression. In one study^20^, the authors demonstrated that saliva analysis can be used to verify the progression of PE, since the authors verified that TNF-α concentration was low at the beginning of PE and increased as it progressed. In the present study, a similarity to the previously mentioned study was observed: the cytokine in question was low in the MP group and increased in the PI implant group. Thus, the TNF-α biomarker was highlighted as a potential tool for the prognosis and progression of PE and PI.

An important aspect of our study related to saliva concentration was discussed in a previous study^30^. The authors observed that total salivary cytokines may represent only a fraction of the total content in saliva and that cytokines can be negatively affected (diluted) by salivary components (mucin), which decreases the detection power of the ELISA assay. Another study showed that mucin had already been found to be increased in saliva in cases of chronic PE compared to periodontally healthy cases.^31^ The literature showed that, when using ELISA for salivary analysis, a correction must be made by the protein such that the test can be considered reliable.^32^ This concern over the influence of salivary protein was considered in our study, since the concentrations of the cytokines were normalized by the salivary protein to attempt to decrease the salivary viscosity effect at the collection time points (T1 and T2).

In addition to the absence of significant differences in the salivary concentration of IL-1β, IL-10 TGF-β and RANK/OPG between T1 and T2, it is worth noting that the hypothesis of this study was finding increased levels of IL-1β, IL- 10, TGF-β and RANK/OPG in the GNTP group compared to the GTP group, especially in individuals who developed PI. However, one study has also observed that the salivary levels of this biomarker are not able to differentiate MP from PI.^33^ This result may have been obtained because of the difficulty in detecting cytokines in PID and PE. Due to the periods of activity and inactivity of these pathologies, at low concentrations, the biomarkers may have an increase or decrease in their release in saliva, FSPI and gingival crevicular fluid.^34^

MMPs have been studied for their ability to cleave components of the extracellular matrix. In particular, MMP-2 has an important relationship with PID because it is able to cleave collagen type 1, which is an abundant component in gingival conjunctive tissue, is linked to the monitoring of collagen degradation and has been associated with tissue destruction in chronic PE.^35^ It was demonstrated that there were no differences in biomarkers salivary concentration between PE and periodontal health.^36^ ^,^ ^37^ This finding reinforces the findings of our study since it diminishes the influence of PE in the results, as the sample in this study had individuals who did not manifest PE at T1 but manifested PE at T2. Due to the important relationship with collagen degradation and scarcity of studies on salivary MMP, we emphasize that it should be used in future studies to better understand the relationship with PID.

Thus, the quantification of salivary markers was considered a promising diagnostic tool for the understanding, prevention and progression of PE and PID.^38^ For future research, it is important to note that, together with cytokine analysis of salivary glands, this approach can greatly benefit the diagnosis because observing a high concentration of a proinflammatory cytokine may produce increased risk of developing PE and PID before its clinical signs of activity are exacerbated.^39^

## Conclusion

This study showed a beneficial role of TMPP in maintaining the balance of periodontal and peri-implant clinical condition and that, in the absence of TMPP, the salivary concentration of TNF-α increased. Additionally, the increased salivary level of TNF-α was associated with worse peri-implant clinical condition. Thus, TNF-α may be considered a biomarker of PID, but new studies in different populations and with different designs are needed to clarify this cytokine’s role in peri-implant diagnosis and progression.
